# Nursing in the United States

**Published:** 1916-05-13

**Authors:** 


### THE MATRONS' AND SISTERS' DEPARTMENT.

### II. NURSING IN THE UNITED STATES.

### STATE REGISTRATION IN AMERICA.

WE have great pleasure in publishing the interesting answers given by Dr. Hurd, of Baltimore, to the following questions which we put to him on matters relating to State Registration in the United States of America and its effects.

**Has Registration Protected the Uniform?**

QUESTION: *Has registration made it impossible or illegal or a punishable offence for anyone but a registered nurse to wear a uniform either identical with, or similar to, those worn by trained nurses in the United States or any portion of them?*

DR. HURD'S ANSWER: I know of no State where it is impossible or illegal or punishable for (anyone but) a registered nurse to wear a uniform identical with or similar to that of a trained nurse.

**Morality, Efficiency, and Acceptability.**

QUESTION: *Has registration made nurses more moral, more honest, more efficient in their duties, and more acceptable to their patients?*

DR. HURD'S ANSWER: In reply to your question as to whether registered nurses have been more moral, more efficient, and more acceptable, I can only say that in my judgment all persons are improved by kindly oversight, proper treatment, educational advantages, and stimulation to greater effort. In America, crimes among nurses due to immorality, dishonesty, and inefficiency are almost invariably traced to the uneducated woman, unfitted mentally and morally to be a nurse, who has slipped into the profession through a poor training school. If registration and inspection exist such persons can be dealt with; if they are not provided, it is impossible to prevent them from preying upon the public.

**The Spirit of Florence Nightingale.**

QUESTION: *Have those who are registered acquired the spirit of Florence Nightingale to a greater extent than non-registered nurses?*

DR. HURD'S ANSWER: I have no hesitation in saying that the better educated a nurse is and the more carefully trained the more thoroughly will she be imbued with the spirit of Florence Nightingale. From my recent reading of the life of Florence Nightingale, I perceived that she did not believe that there was any virtue in incompetence, ignorance, and lack of common-sense. To put your question another way, I would inquire, is the ignorant nurse likely to be more moral, more honest, and more efficient than the educated one?

**Higher Fees.**

QUESTION: *Are registered nurses less inclined to demand heavy fees or has registration tended to increase the demand for high fees and to raise the fees paid to nurses engaged in private work?*

DR. HURD'S ANSWER: In the matter of high fees, I cannot speak for other States, but I know that in Maryland there has been no increase in the fees of trained nurses during the past twenty-five years.

**Registration Helps Smaller Hospitals.**

On this point Dr. Hurd writes: A friend of mine informs me that in his State the general status and efficiency of poorly trained nurses has been greatly improved by registration, and a nurse of the right sort who fails in her examinations thus finds out her weak points, and is encouraged by the examiners to study further or to take a post-graduate course before seeking to take another examination or to receive an endorsement. Such a registered nurse has a much better standing before the public, and in fact a good graduate of a small hospital may, through registration, obtain a recognition of her ability which she could not enjoy without registration.

**An Advantage to the Public?**

On this point Dr. Hurd writes: Registration also is a great advantage to the public, because it furnishes the means of discrimination between ability and incompetence and separates qualified and upright nurses from unqualified and unscrupulous ones.

